# Camping in urban parks as a public health-oriented therapeutic landscape experience accomplished through self-transformation

**DOI:** 10.3389/fpubh.2026.1794375

**Published:** 2026-05-12

**Authors:** Qingyan Huang, Xiaohua Wang, Pengtao Wang

**Affiliations:** School of Tourism, Xi'an International Studies University, Xi'an, China

**Keywords:** camping, public health, self-transformation, SHAP analysis, therapeutic landscape

## Abstract

Driven by growing public health awareness, individuals are increasingly seeking to break free from daily routines and engage in camping activities within urban parks as a strategy for enhancing well-being. This study investigates the mechanisms through which camping in urban parks contributes to campers' health and well-being, with a particular focus on its role as a public health intervention. We critically reassess the concept of “therapeutic landscapes,” which has often overemphasized material aspects while underestimating the significance of subjective perception. Using qualitative methods, including participant observation and semi-structured interviews with 21 campers, we examine the therapeutic dimensions of camping. Additionally, questionnaire data from 338 samples collected in Xi'an, China (2024–2025) were analyzed using SHapley Additive exPlanations (SHAP) to explore the relationships between environmental factors, individual perceptions, and therapeutic outcomes. The results indicate that camping in urban parks functions as a therapeutic experience and serves as a proactive public health strategy through facilitating self-transformation. The therapeutic process involves a transformative journey, beginning with escape from daily stressors, progressing through reflective introspection, and culminating in the reconstruction of life meaning. SHAP analysis reveals that therapeutic outcomes are significantly influenced by individual perceptions and environmental features, underscoring the relational nature of health promotion in such settings. These findings contribute to the expanding literature on therapeutic landscapes and public health, offering a valuable framework for designing nature-based health interventions and sustainable leisure practices in urbanizing societies.

## Introduction

According to the Research on China's Camping Industry and Analysis of Model Enterprises in 2022–2023, the number of enterprises in China's camping industry had surpassed 201,000 by September 2024, with the core market expected to reach 248.32 billion yuan by 2025 (1). Camping has become a significant form of outdoor recreation in urban China (2, 3). We observe that participants conceptualize camping as an escape experience, a family vacation, a lifestyle, or a liminal space as a gateway to eudaimonic happiness—characterized as a sense of purpose and meaningful, positive engagement with life that arises when one's life activities are congruent with deeply held values even under conditions of adversity (3, 4). Critically, despite camping being a popular association with healing activity that enhances self-transformation and well-being (5–7), empirical research remains underdeveloped regarding how these health outcomes manifest.

Despite the growing camping market, research on the relationship between camping and health remains limited (8). Camping provides a unique experience that exceeds the experience of nature and social interaction (9). This distinctiveness allows modern campsites to facilitate escape, rejuvenation, and restoration. Dunkley further conceptualizes camping as a therapeutic landscape—settings reputed for enabling healing, health, and well-being (10). This framework elucidates how camping generates health through escape, self-transformation, and meaning-making (11). However, existing literature primarily focused on isolated aspects like motivations (12, 13), embodied experiences (14), natural benefits (15), psychological benefits (16), campsite attributes (17), medical treatments (18), personal and social development (19), and relationship maintenance (20, 21), neglecting the dynamic co-creation process of therapeutic experience formation between campers and campsites.

As such, we apply the therapeutic landscapes framework to Chinese camping, tracing the camper-site interactions that co-create therapeutic experiences. Using interviews and questionnaires collected in Xi'an, China. We specifically aim to address the following research questions: (1) Why and how do participants organize camping as therapeutic landscapes? (2) How do campsites and campers collectively contribute to therapeutic experience? In this paper, we make a self-transformation analysis of co-created therapeutic experiences within the context of camping.

To answer these questions, this research innovatively applies the therapeutic landscapes framework to the context of contemporary urban camping in China, a phenomenon receiving increasing attention but with limited empirical exploration regarding its therapeutic dimensions. Our findings are expected to contribute significantly to understanding the mechanisms through which camping in urban parks fosters well-being, offering practical insights for promoting nature-based health interventions and sustainable leisure practices in rapidly urbanizing societies.

**Figure 1:**
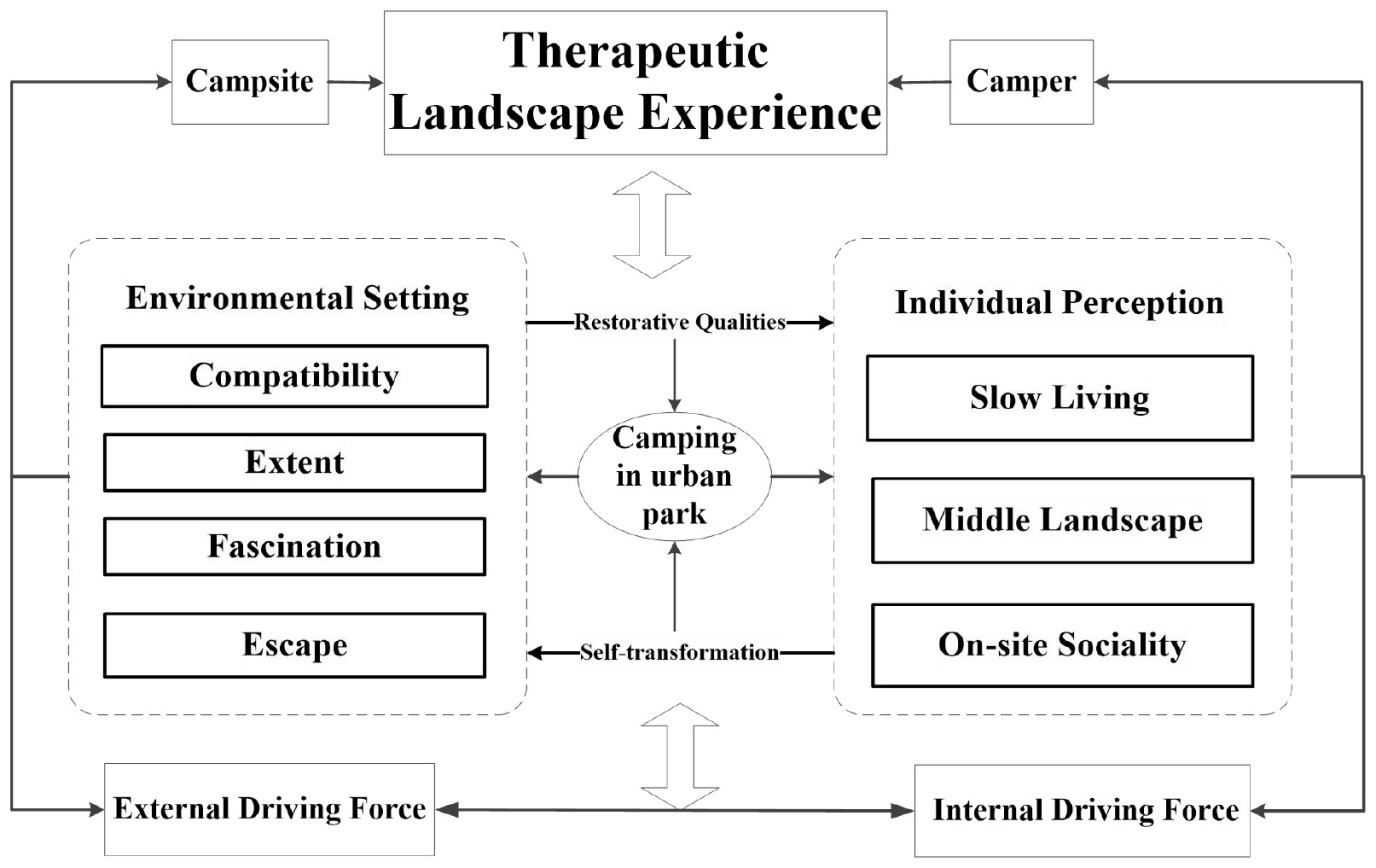
The process of achieving therapeutic landscape experience through camping.

This study demonstrates that camping in urban parks functions as a therapeutic landscape primarily through facilitating a process of self-transformation. Therapeutic outcomes are not delivered by the environment passively but are actively achieved by campers navigating a journey from seeking escape, through engaging in profound reflection within the liminal camping space, toward reconstructing a sense of life's meaning. This process is fundamentally relational, dependent on the synergy between specific environmental qualities of the campsite and the camper's perceptions, agency, and transformative practices (Figure 1).

## Literature review

### Delineating the therapeutic landscape of camping

Wilbert Gesler first introduced the therapeutic landscape at the 1990 International Medical Geography Symposium in Norwich (22), and then Gesler defined it as “a geographic metaphor for aiding in the understanding of how the healing process works itself out in places” (10). Later, Gesler (23) expands it to “the physical and built environments, social conditions, and human perceptions combined to produce an atmosphere which is conducive to healing.” Gesler's attempts at categorizing three strands—namely, physical, social, and symbolic environments. He explains that “The physical environment strand of therapeutic landscapes has two components: natural and human-made or built environments. The social environment is informed by structuralism, also referred to as materialism or political economy. Symbolic environments are created by the mind and involve one's thoughts and beliefs (24).” The therapeutic landscape concept provides a framework for a substantive analysis of various settings that contain therapeutic qualities, ranging from natural environments and traditional, iconic places (25) to healthcare-related sites (26) and everyday places (27).

Inspired by the concept of therapeutic landscapes, Conradson (28) introduces the idea of therapeutic landscape experiences, where outcomes are predetermined but emerge through an individual's active engagement within a particular socio-natural-material environment. Edwards (29) highlights that therapeutic landscape experiences may simultaneously involve feelings of both detachment from and reconnection to everyday life. Bell et al. (30) underscore the significance of embodied experiences in therapeutic landscapes, pointing out that sensory elements such as visual, auditory, and gustatory stimuli enrich these experiences. Building on these insights, our study investigates how campers embody the ethos of “back in nature” and experience a sense of removal from everyday routines.

Camping was a prominent focus of leisure research in the 1960s and 1970s (7); however, it has since received limited scholarly attention, with the exception of a recent comprehensive study by Garst et al. (9). Timothy and Teye (31) note that camping is one of the accommodation types derived from the temporary sheltering practices of armies, but is now recognized as a form of leisure and recreational activity. Blichfeldt and Mikkelsen (32) define camping as the act of setting up tents in natural settings, serving as a financially viable accommodation choice. Garst et al. (9) point out that camping encompasses more than just accommodation, integrating a range of experiences that include recreational, aesthetic, and spiritual aspects. Kim et al. (33) emphasize that camping can promote personal growth, enhance the quality of life, and contribute to overall well-being. Hao et al. (34) point out that camping serves as a powerful and holistic therapeutic activity that allows urban dwellers to escape the stress and routine of daily life for a short period of time and to construct an ideal state of life within a symbolic natural and man-made context. Campers' conspicuous leisure behavior positively affected their subjective well-being, and leisure involvement moderated the relationship between conspicuous leisure behavior and subjective well-being (35).

Despite limited empirical research exploring camping's meanings within therapeutic landscapes (6, 36). Morrow et al. demonstrate that camping serves as a tool to promote general health and well-being in both clinical and non-clinical young adult populations (5). Thus, camping in urban parks, which are special spaces with therapeutic landscapes, may effectively prevent anxiety and depression (6, 34). Building on this, Huang et al. (14) devise a conceptual model illustrating how therapeutic experiences are created through embodied interactions. Crucially, as Hassell et al. (12) emphasize, these experiences fundamentally highlight “the importance of the people-natural environment relationship”. However, less attention has been paid to the important role camping in urban parks plays in healing, restoring, and maintaining human health.

### The therapeutic quality of camping from a transformative perspective

Previous studies show that camping has transformative effects on individuals (37–39). Dai et al. (40) note that camping fosters both a connection to nature and complex social dynamics among participants, which have the capacity to induce positive shifts in individuals' thoughts and perspectives. Research indicates that campers experience improvements in psychological well-being (e.g., reduced stress, enhanced mood) (6), attitude toward self-concept (41), and self-perceived (14). Ayazlar and Ayazlar argue that campsites are perceived as liminal spaces—where everyday societal boundaries and structures are temporarily suspended or dissolved, enhancing self-awareness and enabling eudaimonic happiness (3). Similarly, Jiásek et al. (19) specify that camping contributes in unique ways to the spiritual dimension of one's life. Missing in these studies is attention to camping as a self-transformation activity in order to pursue well-being. In particular, it remains unclear how this pursuit unfolds in a set of transformative qualities embodied in the camping experience.

“Self-reflection” is a central topic in contemporary research on self-transformation. Hassell et al. (12) find that campers are motivated to camp because it contributes to an image they desire for themselves. Increasingly, scholars recognize that camping provides special opportunities for self-reflection. Wearing and Deane (42) suggest that camping, like a journey of the adopted self, allows individuals to self-verify aspects of their ought or ideal self. Heintzman (43) observes that camping environment offers peace, tranquility, silence, and freedom from disturbances, creating essential conditions for self-reflection and solitude absent in daily life. Preliminary findings suggest that self-reflection plays a crucial role in facilitating psychological and emotional recovery, reconstruction, and enhanced overall well-being (44).

Although existing research has not yet fully elucidated the mechanisms by which camping's therapeutic functions are realized through the theory of transformative experiences, the emphasis within transformative experiences in promoting self-reflection and consciousness awakening aligns with the need to develop camping therapeutic theory. This development aims to enhance individual cognition and achieve long-term therapeutic value, thereby providing theoretical space for the innovation of camping healing. Camping offers a supportive and stimulating environment conducive to self-discovery and emotional renewal, both of which are critical components of transformative experiences (45). The self-transformations resulting from camping experience can be complete or partial, isolated or systemic, and short or long-term (46). This perspective is crucial for comprehending the therapeutic effects of camping.

## Methodology

**Figure 2:**
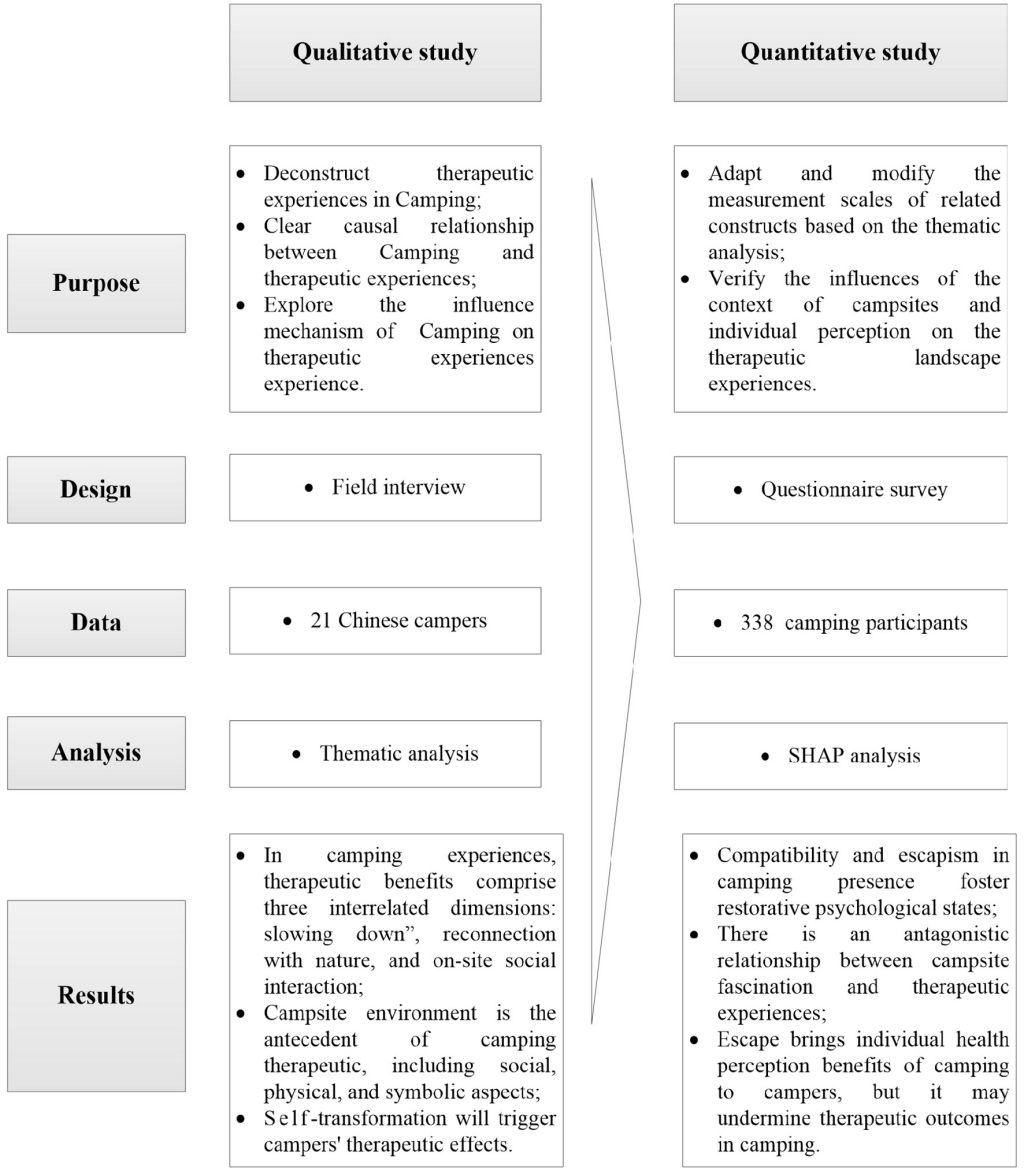
Overview of mixed-methods research approach.

Given that the experience of therapeutic landscapes is inherently subjective and that the therapeutic benefits derived from camping differ among individuals, we document participants' interpretations of their camping experiences while also providing our analysis of the underlying messages conveyed by these experiences. We aim to integrate campers' subjective experiences and personal narratives into our conceptualization of camping as a therapeutic landscape. Therapeutic landscape experiences have not been adequately explored in the Chinese context. We are applying a mixed method of quantitative and qualitative design in explaining why and how camping can be a therapeutic landscape experience. The mixed method design can improve the external and internal validity of the research (47). An overview of our study design is presented in Figure 2.

Specifically, the mixed-methods approach consists of two parts: First, qualitative research, following an explanatory paradigm, explored the dimensions, antecedents, and consequences of campers' therapeutic landscape experience in the camping context. Second, quantitative research, following an empirical paradigm, tested the influences of campsites' contextual features and individual perception on therapeutic landscape experiences. Although the samples differ across the two methods, they were selected from appropriate contexts, and our diverse sample sources helped reduce common method bias (48).

### Interviews and data analysis

We applied a qualitative methodological approach using semi-structured interviews to understand campers' experiences, while necessarily offering our own interpretation of the underlying messages that their experiences convey. Interviews were conducted in Xi'an, one of China's top ten cities for camping (49), located in central Shaanxi Province, northwest China. As of January 2024, the relevant government departments of Xi'an, in collaboration with “Xiaohongshu” (a popular Chinese app and a leader in China's UGC e-commerce sector, featuring a large amount of user-authentic review content that attracts a large number of young users (50), have launched a series of camping development support measures, such as the “Exclusive Page for Xi'an Camping”, the “Camping Academy”, and “Providing Information Support for the Development of Local Camping Enterprises”. To achieve a more profound understanding of the interviewees, we engaged in camping experiences with some of them at various urban parks. These activities took place in May, July, and October 2024, as well as in March 2025. Specifically, we visited three renowned campsites in Xi'an: Heguang Farm, Chang'an Tang Village, and Tianqiao Lake International Camping Site.

The interviews were conducted in Chinese and comprised the following: can you tell us something about your experience on this camping? How did you feel during the camping? What personal significance does camping in this urban park hold for you? Do you feel camping here allows you to be a different version of yourself, even temporarily? Unstructured conversations prevailed during the interviews to allow campers to illustrate their personal experiences. We interviewed 21 Chinese campers (Table 1). Pseudonyms were used to protect respondent anonymity, such as “LY1”. Each interview was 30–60 min long and was audio recorded and transcribed. Eleven men and 10 women participated, aged 20–70.

**Table 1:** Basic information of respondents.

Pseudo name	Age	Frequency of camping (times)
LY1	50–60	20+
LY 2	30–40	20+
LY 3	40–50	10
LY 4	30–40	6
LY 5	30–40	3
LY 6	30–40	10
LY7	40–50	20+
LY 8	20–30	5
LY 9	30–40	3
LY 10	30–40	10
LY 11	30–40	4
LY 12	40–50	20+
LY 13	20–30	10
LY 14	70–80	20+
LY 15	40–50	20
LY 16	40–50	10
LY 17	40–50	20+
LY 18	30–40	20+
LY 19	60–70	2
LY 20	30–40	3
LY 21	20–30	4

**Figure 3:**
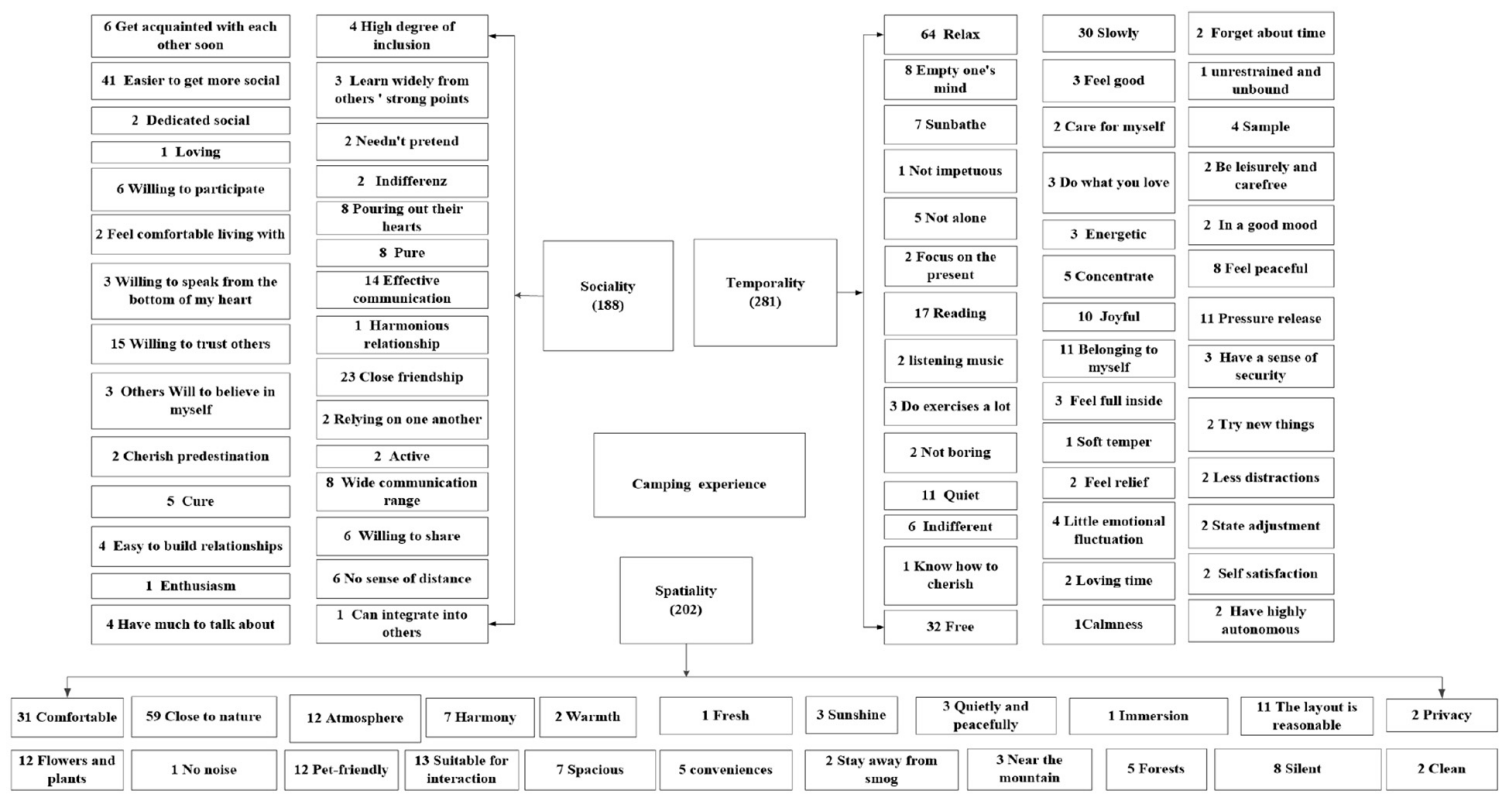
Camping experience.

A key part of the data analysis was to examine these interviews in the context of the therapeutic landscapes concept to identify how camping acted as a therapeutic landscape experience. We applied thematic analysis to the interview data to identify the experience of camping and the components of therapeutic landscapes. This method entails searching a data set to identify, analyze, and report a repeated pattern (i.e., theme), which is a patterned response or meaning that is closely related to the research problems and reflected in the qualitative data (51). Key themes found included: campsite environment and atmosphere, associations with and attachments to camping. These themes were subsequently analyzed through the lens of the therapeutic landscapes concept, particularly in relation to how the camping was formed and evolved as a therapeutic landscape, the different elements that informed this formation and evolution, and how these elements and processes affected campers' health and well-being. This led to the emergence of the key social, physical, and symbolic elements, and how these contributed to form and evolve camping as a therapeutic landscape experience. A substantial volume of qualitative textual data was generated through the research. To systematically analyze the core dimensions of camping experiences, thematic analysis was conducted using NVivo 11 Plus software. This process yielded three empirically grounded thematic constructs: temporality (188 co-constructed reference points), spatiality (281 co-constructed reference points), and sociality (202 co-constructed reference points). These themes collectively inform an exploration of how camping experiences contribute to health promotion, as illustrated in Figure 3.

### Questionnaire and data analysis

As Atkinso indicates that the therapeutic mechanisms vary with different contexts and individual (52). We adopted the method of a questionnaire to empirically examine the influences of the context of campsites and individual perception on the therapeutic landscape experiences. In this study, we use previously validated scales to ensure the validity and reliability of measurement. The large body of literature in the area of perceived environmental restorative quality has fully demonstrated the influence of place context on therapeutic outcomes (53). Chen et al.'s (54) study provides a Perceived Destination Restorative Qualities scale (PDRQS) for the Chinese context with four dimensions, namely compatibility, extent, escape, and fascination. Concomitantly, for the variable of individual perception in this paper, three items were used to measure individual health perception benefits. Three items—improved condition, prevention of a worse condition, and realization of a psychological state—were used to measure individual health perception benefits (55), and previous research has confirmed that this scale is both suitable and reliable for assessing tourists' health perceptions regarding outdoor recreational activities (56). As a result, this study will investigate how the perceived restorative quality of campsites (measured via the PDRQS scale) and individual health perception affect the therapeutic landscape experiences of camping. The relevant meaning of each indicator is shown in Table 2. In this study, a single-item self-report measure was employed to assess the perceived therapeutic benefits of the camping intervention: “Overall, the camping experience has produced significant improvements in my physical and mental well-being.”

**Table 2:** Research element breakdown table.

Research elements	Research indicators	Indicator description	Naming of research indicators
Restorative qualities of campsite environment	Compatibility	Consists of the degree to which the place is in line with the individual's interests	*X*1
	Extent	The extent to which parts of a place are perceived as belonging to a whole, and there is a wealth of stimuli that invite exploration of the place, including aspects such as openness, mobility, or care for the environment	*X*2
	Fascination	The ability of the environment to attract the attention of the person without effort.	*X*3
	Escape	The extent to which a place allows the person to distance themselves from daily problems and concerns	*X*4
Individual health perception benefits of camping	IMP	Improved condition	*X*5
	PREV	Prevention of a worse condition	*X*6
	PSYC	Realization of a psychological state	*X*7

Our questionnaire was developed in three parts: (1) scale of perceived environmental quality, (2) scale of health perception benefits, and (3) sociodemographic variables. To study the therapeutic mechanisms of camping, a questionnaire was administered on May, July, and October 2025, at three famous campsites in Xi'an, namely Heguang Farm, Chang'an Tang Village, and Tianqiao Lake International Camping Site. The study collected 338 valid questionnaires from participants who were all Xi'an residents, with participant ages ranging from 16 to 73 years. Table 3 provides an overview of the sociodemographic data for the study participants. In the following sections, we will discuss how the therapeutic landscape experience unfolds in the camping in urban parks in Xi'an.

(1) Scale reliability and validity test

**Table 3:** The information of the questionnaire sample.

Demographic characteristics		Sample size	Percentage (%)	Demographic characteristics		Sample size	Percentage (%)
Gender	male	139	41.12	Occupation	Freelancer	11	3.3
	female	199	58.88		Private owner	4	1.2
Marital status	unmarried	88	26.04		Workers	3	0.9
	Married without children	37	10.95		Pupil	46	13.6
	Married with children	213	63.01		Homemaker	2	0.6
Education	Less than high school	73	21.60		Retirees	2	0.6
	High school	38	11.24		Other	11	3.3
	Junior college	29	8.58	Camping experience	1 time	32	9.5
	Undergrad	103	30.47		2 times	53	15.7
	Master's and above	95	28.11		3 times	80	23.7
Personal monthly income (Yuan)	2,000 and below	26	7.7		4 times	37	10.9
	2,001–5,000	47	13.9		5 and more times	136	40.2
	5,001–10,000	131	38.8	Last camping stay time	No overnight stay	94	27.8
	10,001–20,000	106	31.4		One night	178	52.7
	20,001+	28	8.3		Two nights and more	66	19.5
Occupations	Government clerk	16	4.7	A companion from a recent camping trip	Oneself	3	0.9
	Professional/cultural and educational technicians	86	25.4		Family members or relatives	163	48.2
	Business people	13	3.8		Friend	154	45.6
	Private/foreign company employee	141	41.7		Unit organization	7	2.1
	Service/sales people	3	0.9		Professional camping team	11	3.8

First, Cronbach's α value was used to test the reliability of the scale. The results showed that the Cronbach's α values for the scale is 0.928, and the seven factors are 0.831, 0.726, 0.781, 0.803, 0.824, 0.886 and 0.846, respectively, all exceeding the 0.7 threshold, indicating good reliability of the scale. Convergent validity is evaluated by checking the values of all the factor loadings for individual items (>0.50) and the values of average variance extracted (AVE > 0.50). As shown in Table 4, the factor loadings for the 42 items are between 0.592 and 0.875. The AVE values for compatibility, extent, escpe, fascination, IMP, PREV, PSYC were 0.5197, 0.5669, 0.5017, 05109, 0.5409, 0.6311 and 0.6079, respectively, suggesting sufficient convergent validity.

**Table 4:** Result of reliability and validity test.

Research indicators	Item	SFL	AVE	CR	Cronbach's α
*X*1 (Compatibility)	1. The campsite I visited was consistent with who I am	0.794	0.5197	0.8274	0.831
	2. Everything I saw at the campsite goes well together	0.6			
	3. Campsite suits my personality	0.771			
	4. I have a sense of oneness with campsite	0.621			
	5. Everything I saw at campsite belongs there	0.757			
	6. Campsite was in harmony with its natural surroundings	0.655			
	7. Campsite was my kind of place	0.703			
	8. The things I like to do can be done at campsite	0.838			
	9. The things I could do at campsite were things I was looking forward to before the trip	0.713			
*X*2 (Extent)	10. There was a variety of things to do at campsite	0.872	0.5669	0.8667	0.726
	11.I could do many things at campsite	0.71			
	12.I did different things in different areas at campsite	0.732			
	13.Campsite was large enough to allow exploration in many directions	0.713			
	14. Campsite allowed me to explore extensively	0.725			
*X*3 (Fascination)	15. For me, camping was a captivating experience	0.644	0.5017	0.8338	0.781
	16. My attention is drawn to many interesting things about campsite	0.774			
	17. There was much to explore and discover at campsite	0.707			
	18. I found campsite fascinating	0724			
	19. Being at campsite makes me wonder about many things	0.686			
*X*4 (Escape)	20. At campsite, I could forget about my obligations	0.724	0.5109	0.8783	0.803
	21. At campsite, I felt that I was away from everything	0.592			
	22. When I was at campsite, I felt free from my daily routine	0.701			
	23. At campsite, I felt free from all the things that I normally have to do	0.61			
	24. Being at campsite, I felt as if I was in surroundings different to my normal living environment	0.749			
	25. When I was at campsite, I did different things from when I was home	0.829			
	26. Campsite was very different from my daily environment	0.776			
*X*5 (IMP)	27. Camping improves my overall fitness	0.726	0.5409	0.8248	0.824
	28. Camping improves my overall health	0.751			
	29. Camping improves muscle strength	0.697			
	30. Camping improves my physical flexibility	0.766			
*X*6 (PREV)	31. Camping reduces my chances of developing diabetes	0.758	0.6311	0.8948	0.886
	32. Camping reduces my chances of weight gain	0.875			
	33. Camping reduces my chances of having a heart attack	0.809			
	34. Camping reduces my chances of premature death	0.812			
	35. Camping reduces my number of illnesses	0.708			
*X*7 (PSYC)	36. Camping causes me to appreciate life more	0.748	0.6079	0.9154	0.846
	37. Camping causes me to enjoy life more	0.836			
	38. Camping causes me a sense of self-reliance	0.729			
	39. Camping causes me a sense of higher self-esteem	0.794			
	40.Camping makes me more aware of who I am	0.824			
	41. Is connected to other positive aspects of my life	0.779			
	42. Camping causes me to be more satisfied with my life	0.741			

The content of the measured items is derived from established scales with high content validity. Secondly, using Amos 23.0, a confirmatory factor model is established, and the measurement model's fit indices are as follows: χ^2^/df = 1.700, GFI = 0.890, AGFI = 0.863, CFI = 0.918, TLI = 0.906, IFI = 0.920, RMSEA = 0.049. All these indicators met the standards proposed by West et al. (57).

(2) SHAP analysis

Unlike traditional statistical methods that rely on linear assumptions, this study employs Random Forest Regression as the baseline machine learning model to facilitate model fitting prior to SHAP value analysis. Random Forest is an ensemble learning method based on decision trees. It enhances model accuracy and stability by constructing multiple decision trees and aggregating their predictions, demonstrating robust resistance to overfitting and strong adaptability to high-dimensional data. Following optimization via grid search and cross-validation, the final hyperparameters of the Random Forest model are presented in Table 5.

**Table 5:** Random forest model hyperparameter settings.

Parameter	Setting	Description
n_estimators	8,000	Number of decision trees; increases model stability through a greater number of trees.
max_depth	6	Maximum depth of each decision tree; controls model complexity.
min_samples_split	15	Minimum number of samples required at a leaf node; enhances model generalization.
min_samples_leaf	8	Minimum number of samples required at a leaf node; enhances model generalization.
max_features	‘sqrt'	Number of features randomly considered for each tree, set to the square root of the total features.
random_state	1	Random seed; ensures reproducibility of results.

This study utilizes 5-fold cross-validation to evaluate model performance. The specific steps are as follows: (1) The entire dataset is randomly partitioned into 5 equal-sized subsets. (2) In each iteration, 4 subsets are used as the training set, and the remaining 1 subset serves as the validation set. (3) This process is repeated 5 times, with a different subset used as the validation set each time. (4) The mean and standard deviation of the evaluation metrics across the 5 validation runs are calculated as the final performance indicators. The cross-validation process employs a random seed of 1 to ensure result reproducibility. The following metrics are used to assess model performance:

(3) Root Mean Square Error (RMSE),

$$\begin{array}{l} \text{RMSE =}\sqrt{\frac{1}{n}\overset{n}{\underset{i=\;1}{\sum }}{\left(y_{i}-{ŷ}_{i}\right)}^{2}} \end{array}$$

This metric measures the average deviation between predicted and actual values; smaller values indicate more accurate predictions.

(4) Coefficient of Determination (R-squared, R^2^)

$$R^{2}=1-\frac{\sum_{i=1}^{n}{\left(y_{i}-{\hat{y}}_{i}\right)}^{2}}{\sum_{i=1}^{n}{\left(y_{i}-y\right)}^{2}}$$

This metric quantifies the proportion of variance in the dependent variable explained by the model. Its value ranges from [0, 1], with values closer to 1 indicating better model fit.

Following 5-fold cross-validation, the Random Forest model achieved a mean RMSE of 0.4730 and a mean *R*^2^ of 0.4436 on the training sets. When trained on the full dataset, the model achieved an RMSE of 0.5917629766025175 and an *R*^2^ of 0.7651081162372063, indicating strong predictive capability and providing a reliable explanatory basis for subsequent SHAP analysis. The SHAP value is the Shapley value for a feature value, which is calculated using the conditional expected value function of the machine learning model (58). SHAP considers all possible combinations of features when calculating the contribution of each feature to the prediction. The data were statistically analyzed using SPSS 26.0 software to obtain the average values of each variable. Before conducting the SHAP (SHapley Additive exPlanations) analysis, the scores of multiple items of each variable collected from the questionnaires were averaged using SPSS 26.0 software, and the average value of each variable was used as the feature value for subsequent analysis. Python 3.13 software was applied to calculate the SHAP value to explain and evaluate the contribution of each feature variable to the health perception outcome.

The dependent variable *Y* = *f* (*x*) is “therapeutic outcome of camping”, defined as the magnitude of change in an individual's overall health status following a single camping intervention. To prevent target leakage, predictor features (*X*) and the target variable (*Y*) were drawn from non-overlapping questionnaire modules. This variable originates from the 43rd item of the questionnaire, measured using a 7-point Likert scale (1 = strongly disagree, 7 = strongly agree). This item aims to capture respondents' overall evaluation of the therapeutic effect of their camping experience, with the specific statement: “Overall, the camping experience has produced significant improvements in my physical and mental well-being.” This variable serves as the dependent variable (*Y*) for the Random Forest model, used for fitting and prediction. The Random Forest regression model constructed in this study can be formally expressed as: *Y* = *f*(*X*) + ε

*Y* is the dependent variable, value measured by the 43rd questionnaire item). {*X*_1_, *X*_2_, *X*_3_, *X*_4_, *X*_5_, *X*_6_, *X*_7_} is the vector of independent features.

*f*(*X*) is the non-linear mapping function learned by the Random Forest regressor.

ε is the random error term.

To facilitate model computation, all variables were standardized using Z-score normalization prior to being input into the model:


$$\begin{array}{l} X_{i}^{scaled}=\frac{X_{i}-\mu_{i}}{\sigma_{i}} \end{array}$$

Where μ_*i*_ and σ_*i*_ are the mean and standard deviation of the *i* feature across all samples, respectively. This standardization eliminates the influence of differing units among variables, ensuring comparability among features during model training.

Upon completion of Random Forest model training, this study employs SHAP (Shapley Additive Explanations) for model interpretability analysis. SHAP values are based on the Shapley value concept from cooperative game theory. By calculating the marginal contribution of each feature across all possible subsets of features and weighting them appropriately, SHAP derives a value for each feature that quantifies its impact on the model output. The formula for calculating SHAP values is:


$$\begin{array}{l} \phi_{i}\left(f\right)=\underset{S\subseteq N\backslash \left\{i\right\}}{\sum }\frac{|S|!\left(|N|-|S|-1\right)!}{|N|!}\left[f\left(S\cup \left\{i\right\}\right)-f\left(S\right)\right] \end{array}$$

ϕ_*i*_(*f*) represents the SHAP value for feature *i*.

*N* is the set of all features.

*S* is a subset of features that does not include feature *i*.

*f*(*S*) is the model's prediction based on the feature subset *S*.

**Figure 4:**
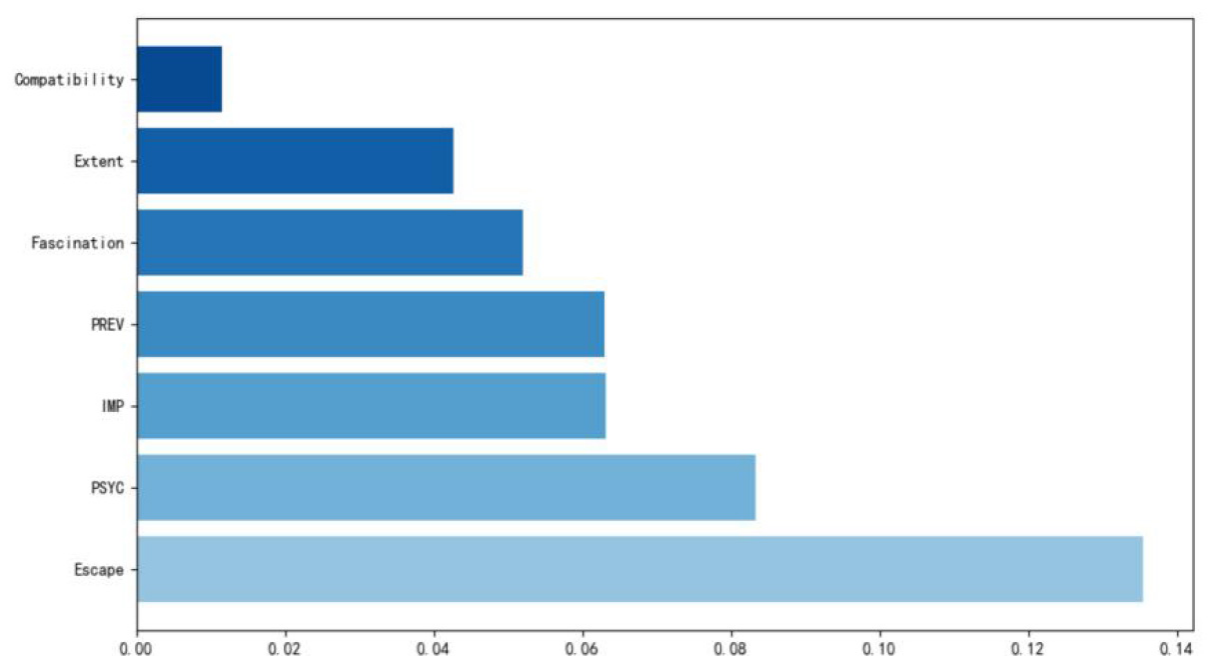
Feature importance in SHAP.

**Figure 5:**
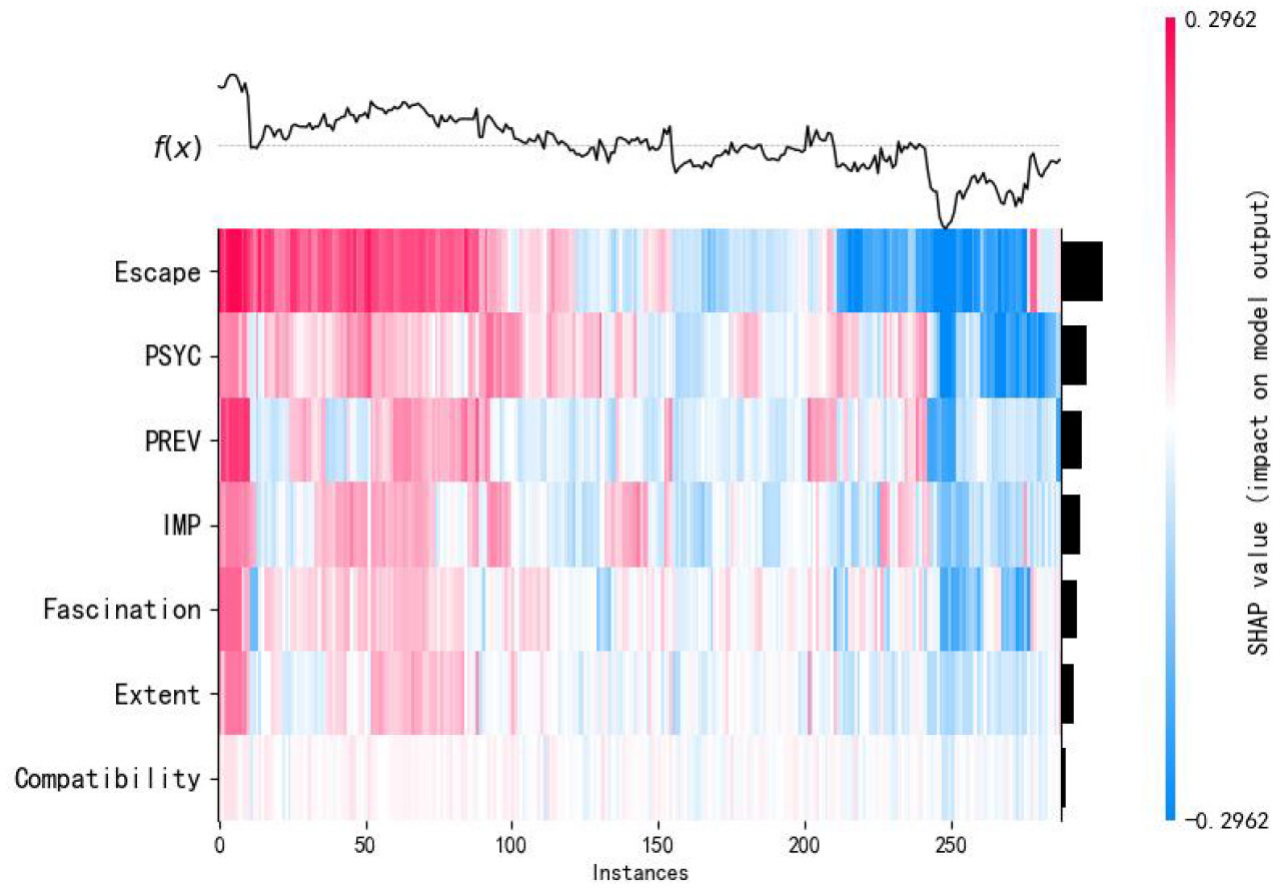
Feature importance plot.

This study utilizes the TreeExplainer algorithm to compute SHAP values. This algorithm is specifically optimized for tree-based models and enables efficient and precise calculation of feature contributions and their interactions. The feature importance was ranked based on the average absolute SHAP values (Mean SHAP value) across all samples. Visual representations, as depicted in Figures 4, 5, highlight the significance of various research indicators on therapeutic effects, providing a comprehensible overview of the findings. In Figure 5, the *X*-axis is essentially the average magnitude change in model output when a feature is integrated out of the model. The features are ordered by the average absolute sum value of their effect magnitudes on the model. The *Y*-axis indicates the feature names in order of importance from top to bottom. The three most impactful variables contributing to therapeutic effects, namely escape, PSYC, IMP, all exhibit values are higher than the mean SHAP value (0.00). Additionally, the following four relatively significant variables are fascination, PREV, compatibility, and extent.

The goal of SHAP is to explain a prediction *f* (*x*) of an instance x by computing the relative contribution of each feature value to the specific outcome (59). Each dot is colored by the value of the feature, from low (blue) to high (red). Black dots represent missing values (60). The effects of Escape and IMP in this study exhibit a bidirectional effect at high values, indicating that excessive Escape and IMP may trigger unstable therapeutic outcomes (both positive and negative). Figure 4 illustrates Fascination and PREV shows a negatively skewed impact, where low values strongly reduce therapeutic outcomes. In contrast, PSYC and Compatibility demonstrate stable positive effects at high values, confirming their critical roles in enhancing therapeutic outcomes. Extent displays inconclusive distributed effects, warranting further study, while Extent and PREV shift from predominantly negative impacts to substantial positive effects when feature values are high.

**Figure 6:**
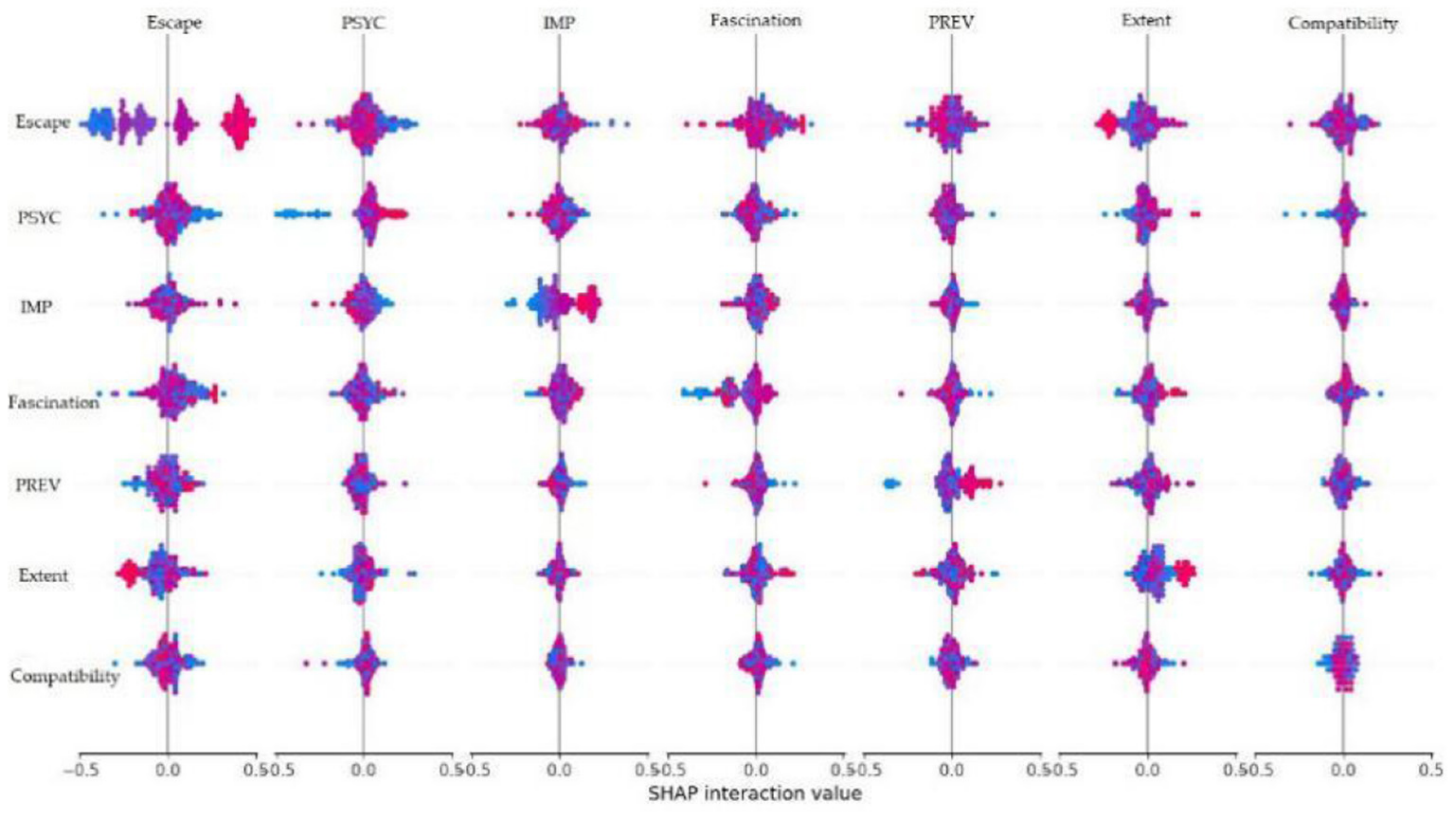
SHAP interaction values analysis.

Based on the SHAP interaction values presented in Figure 6 the key feature interdependencies influencing therapeutic outcomes of camping can be summarized as follows: (1) Compatibility and PSYC display the strongest positive synergy (interaction value ≈ 0.5), indicating that their joint implementation substantially enhances beneficial therapeutic effects. (2) Escape positive reinforcement with PSYC at ≈0.5, negative interactions with Fascination and PREV at ≈–0.5, indicating these combinations may trigger therapeutic outcomes instability. (3) Fascination displays antagonistic relationships with both PREV and IMP, with interaction values ≈–0.5. This suggests that simultaneously high openness with these features counteracts positive effects. (4) Extent exhibits weak interactions overall (near 0.0), confirming its isolated impact observed in previous analyses.

## Results

### What experiences do individuals pursue during camping?

Camping is often associated with freedom (61), development (19), restorative (25), and even eudaimonic happiness (3). When asked “Why do you enjoy camping?” interviewees frequently highlighted relaxation as a dominant response. Our analysis deconstructs what “the camping experience is very relaxing” signifies within therapeutic landscapes, examining its spatial, temporal, and social dynamics. This provides a framework for understanding how such expressions connect to broader therapeutic experiences.

#### Temporality: slow down and an alternative lifestyle

Hassell et al. (12) notice that campers want to escape from busy home lives and fulfill their quest for freedom and well-being at a spiritual level. In our study, the interview reveals that escaping the hectic urban life is a key goal of camping. In China, the 996 and 007 work schedules have become major concerns for individual health (62, 63). The so-called ‘996' schedule requires workers to work from 9 a.m. to 9 p.m., 6 days a week. There is also 007, working 24 h a day, 7 days a week (62). As LY7 explains,

*The most discomforting aspect is not just the 007 or 996 work schedules, but the blurring of boundaries between work, life, and leisure. My mobile phone has become a constant office tool, making it hard to distinguish between professional and personal time*.

A pervasive sense of time-related anxiety has emerged as a shared experience among the public. LY7 notes that extreme busyness gradually erodes individual autonomy and the sense of time, leading him to seek temporary “*points of presence”* to alleviate this anxiety.

LY1′s explanation of his camping hobby provides a compelling interpretation of the idea that camping serves as an escape from the demands of everyday life, fosters a sense of comfort, and ultimately facilitates rejuvenation. He says,

*Camping is more efficient than drinking coffee or traveling; after all, we can enjoy coffee during the week, while traveling necessitates extensive planning and preparation that frequently leads to haste. In contrast, camping provides considerable freedom in terms of time and convenience, typically just an hour's drive away facilitates spontaneous departures and returns*.

For LY11 and other respondents, camping in urban parks represents a lifestyle choice and an effort to escape the demands of daily life, exemplifying what Parkins and Craig refer to as “slow living” (64). As suggested by Parkins and Craig, slow living offers the chance to slow down daily life and manage time and space in ways that are better for personal satisfaction and well-being (64). The accounts provided by our respondents indicate that camping creates optimal conditions for disengaging from the pressures inherent in a hectic lifestyle and temporal constraints, thereby enabling individuals to experience the benefits of temporal autonomy.

#### Spatiality: the middle landscape between the city and the natural environment

An increasing amount of research has underlined the significance of contact with nature and its positive effects on health promotion, such as reducing stress and anxiety, cultivating connections, and facilitating relaxation (65, 66). In Chinese culture, the close connection between the self and the universe is an essential approach to achieving health (67, 68). Our research finds that camping in urban parks, which Tuan (69) describes as a “middle landscape”, offers an intermediary space between wild nature and the metropolis. This setting allows campers to experience an ideal balance between ideals and reality, as LY2 explains,

*My desire for nature is as strong as my desire for the city. What I like most about camping is that it offers a break from my apartment or office while still providing urban conveniences. We choose campsites based on amenities like parking, public restrooms, and play facilities*.

Several points in LY2′s statement are worth noting. The pursuit of camping in urban parks is not about the pleasures of mountains and forests; instead, it aims to create an alternative and heterogeneous “leisure space” by integrating nature and the city.

#### Sociality: on-site social interaction and relationship intensification

Previous studies have considered that real-time, on-site social interactions can promote health and well-being (70). Rihova et al. (71) explain that camping together enables families or friends to socially value co-creation. LY1 explains,

*At home, my family is constantly on their mobile phones, leaving little room for communication. When camping, we set up a tent, have barbecues, and truly engage in calm and meaningful conversations*.

L1′s explanation of camping with his family is the collaborative production of relational value through shared activities. This shift from digital isolation to joint physical activities and communication enables families to co-create relational well-being through intentional engagement. With its inherent social attributes of family communication and interpersonal interaction, the camping experience has evolved into a practice based on blood, relatives, or acquaintances—a distinctive group practice. The communication, division of labor, and cooperation in the process of camping provide a social experience of “on-site exchange” (72), which is transformed into the emotional energy that endows intimate relationships. Camping reunites individuals, family members, and even other close relationships into an organic community.

### How the campsite operates as a therapeutic landscape

As Kearns and Milligan mention, when taking therapeutic landscapes as an analytical framework, all three environments (social, physical, and symbolic) should be employed (22). In the stories provided by our camper respondents, several key factors emerge that are closely associated with the social, physical, and symbolic dimensions of the therapeutic landscape, as shown in Table 6.

**Table 6:** The therapeutic environment at the campsite.

Environment strand	Specific elements	Examples
Physical aspect	Nature Animals Camping gear	Fresh air, mountains, water, plants and flowers, wind, sunrise and sunset, sunshine, sky, grass, etc.) Cats, dogs, etc. Tent light, tea set, coffee maker, stereo, folding chair, etc.
Social aspect	Intimate relationship Social support Human interaction	Family intergenerational relationship, interest relationship, etc. Communication, sharing, games, entertainment, chatting, etc. Tent setting, walking, biking, photo taking, barbecue, etc.)
Symbolic aspect	Rituals Meaning creation Symbolic activities	Setting up camp, boiling tea around the stove, watching movies in the open air, etc. Faith in natural ecology, reflexive thinking, etc. fun activities, etc.

#### Physical aspect

Gesler and Curtis (73) state that therapeutic landscapes include both natural and built elements. Within camping contexts, natural elements play a foundational role: proximity to mountains and rivers enables stress recovery through physiological mechanisms like blood pressure reduction (74). Bell et al. (75) explain that natural elements consistently serve as a central trope within the therapeutic landscapes literature, contributing to the affective, embodied, multisensory alettes of place experiences. These interconnected dimensions collectively create environments conducive to therapeutic outcomes.

Each interviewee clearly describes the meaning of natural elements in the process of the camping experience, and even being close to nature constitutes the pursuit of the authenticity of the camping experience. Although the natural physical aspect of camping environments is not pristine wilderness (76), it reduces temporal and spatial distance between campers and nature, thereby fostering therapeutic connections with the natural environment (77). As LY11 says,

*In nature, breathing the fresh air and looking at the green mountains and rivers of nature, not only purifies the lungs, but also relaxes the eyes*.

Natural elements are considered primitive, even idealized, and objectively existing healing elements (75). Immersed in nature, the campers' attention can be transferred from the living environment, and psychological relaxation and restoration can be obtained from the close contact with nature.

One Health drawing together human, animal and environmental health into a single gaze, under the “one health” turn, animals are increasingly recognized for their impact on human life and health (78). Animal companionship and interaction can stimulate new action potential and provide emotional support and social satisfaction (79). The existence of animals basking in the sun or running everywhere in the campsite caters to the camping subject's imagination of relaxation, leisure, and an optimized atmosphere, and even becomes a unique attraction of the camping space. LY3 describes,

*Just watching the cats and dogs leisurely in the sun, you can feel what is the peace of time. Looking at dogs curled up in the sun, warm and lazy, I really a little envious*.

Nature and animals directly give camping a rich variety of sensory experiences, and refined, quality camping equipment provides an authentic living experience (80). Various forms of decorative equipment are increasingly sought after by campers, which also promotes camping from simple outdoor leisure to being embedded in the life logic of poetic dwelling. Tents, sleeping bag canopy, and other functional equipment to enhance the comfort of the camping experience, audio, atmosphere lamp, coffee machine, tables and chairs, flowers, and other equipment to create a refined, romantic life scene. LY3 mentions,

*I am very happy with the whole camping equipment. It is the curve of workers to save the country, and the ideal life is often out of reach, but through camping, through this equipment, in nature, to create an idealized home is still within reach*.

Camping equipment, by mediating an ideal balance between nature and daily life and facilitating the construction of a temporary “natural home”, reveals that its significance extends beyond mere substantial material existence. Rather, it embodies and supports the ideals of life and the spatial realization of “homing desire” (81), which is the fundamental human longing for belonging, security, and connection. This self-transformation shifts the therapeutic narrative of camping away from pure escape or leisure toward the deeper ontological meaning of fulfilling this core desire. Thus, camping equipment serves as a crucial physical conduit through which this “homing desire” is materially engaged and experientially addressed within the therapeutic context.

#### Social aspect

Gesler and Curtis (73) suggest that the character and quality of social relationships among individuals and within groups are of paramount importance for healing. In the stories our campers share, it is clear that campsites are ideal environments for social interaction. Campsites provide open spaces that foster new social relationships and specific domains that strengthen existing bonds. As LY12 explains,

*Camping together promotes easy interaction. When camping, my family can engage in various activities, communicate effortlessly, and better understand and support each other*.

In addition to the inherent relationships among relatives and friends, urban camping spaces offer a natural and shared environment that fosters high neighborliness. This provides a special way for strangers to interact and form new social relations. LY7 mentions,

*When we go camping, it's common to chat with nearby people. Kids naturally play together, while adults cook around the tent, share meals, or help with group photos*.

The above experience intuitively shows that the camping experience has natural social attributes. A camping site is not only a material space close to nature, but also a place for the production of social relations and the construction of social networks. Social communication and interpersonal support have always been regarded as an important dimension of health promotion. The intimacy of inherent social relations and the expansion of new social relations in the process of camping experience are all important elements that affect individuals to obtain positive emotions and even enhance happiness (82).

#### Symbolic aspects

As Kleinman claims, the symbol system facilitates the healing process by connecting the biophysical and sociocultural worlds through various rituals and treatments (83). LY8 said he enjoys making tea while camping. He mentions,

*I enjoy cooking tea while camping. Tea is nature's gift. I bring walnut or olive carbon, light the stove, and watch the water boil as the tea leaves unfurl. This ritual reminds me of the simple pleasures in life: a pot of hot tea, an old song, family and friends, and watching the clouds drift by*.

Campers create a specific ritual of brewing tea around the stove. For LY8, camping is more than just outdoor recreation; it reflects his vision of an ideal life. As some scholars have noted, tea drinking in its specific environment forms a healing landscape in a non-iconic, localized, and generalized sense.

In the process of camping, rituals similar to tea brewing activities are various and distinctive. For example, a series of camping equipment such as ambient lamps, projectors, coffee machines, sky curtains, and folding chairs is used to create a specific sense of living atmosphere and life rituals, reflecting the pursuit of a healthy and beautiful life in the camping site. In addition to the creation of the ceremony, camping itself has also been given the “natural therapy—a therapeutic approach that leverages exposure to natural environments to elicit physiological relaxation and strengthen impaired immune function” (84) consumer label and symbol value, LY2 is very succinctly summed up,

*Camping offers a classic example of the 20-minute outdoor effect, where spending just 20 minutes in nature can significantly boost mood and energy levels, even without physical exercise. In my view, health and well-being fundamentally involve reconnecting with nature, breathing fresh air, consuming nutritious and green foods, and stepping away from the confines of offices or homes*.

LY2′s understanding and value judgment of the relationship between camping and healing, in essence, regards camping as a holistic state of life. Rather than camping being a life paradigm built around healing, it is better to say that healing is placed in each part of the camping experience. People are healing themselves while also practicing the need for a better life. The symbolic elements included in the camping experience, in the context of social acceleration, re-provide a series of “resonance relations”, that is, interpersonal relations, people and nature, people and matter mutually respond to each other in resonance relations (85).

### Why camping achieves therapeutic effects: self-transformation

#### The subject's proactive engagement in self-transformation

Examining campers' intricate inner states during camping reveals that therapeutic effects emerge from dialectical connections between tradition and modernity, individual experience and social environment. This aligns with existing research framing therapeutic outcomes as relational—dependent on interdependence, interaction, and extension between individuals and their environment (28). Previous studies on therapeutic outcomes have emphasized the agency of participants in the construction process of the therapeutic landscape, but have overlooked the extensive influence of participants' subjective experiences on the therapeutic benefits (86). As LY13 says,

*After experiencing COVID-19, my top priority is now my own well-being. As long as I am happy, that is enough. With such beautiful blue skies and white clouds, why not enjoy them fully?*

LY13 implies that campers do not merely adapt passively to the environment but actively reshape it through their perceptions, experiences, and practices (87). The daily struggles and sense of purposelessness expressed by campers have fueled their strong desire for wellness, driving them to actively seek moments of authenticity and pursue self-release and healing. Campers act on their values and interests, obtain interpersonal care and support from others, and achieve a temporary “poetic dwelling” (88). This dynamic interaction enables campers to actively recalibrate their environment, thereby bridging survival reality and ideal life (89). Consequently, camping's therapeutic effect evolves from sensory relaxation to a deeper, self-directed restoration of holistic health (90).

The therapeutic practice of camping is essentially a typical way for individuals to actively self-care under the real situation of environmental stress and exert an active effect on the environment (91). This implies that the subjects are not passively adapting to the environment but also exerting an active influence and effect due to their perception, experience, and practice (87). Ultimately, it is a geographical phenomenon of positive interaction between humans and the land, conforming to the health mechanism interpretation of “the harmony of heaven, earth, and humans” in Eastern Health Philosophy (92).

#### Self-reflection during the process of escape

In the research tradition of health geography, psychological or physical health issues are often ascribed to the environmental traits of the individual's habitation space (87). Therefore, in the extant studies on the relationship between camping and health, escape has emerged as the core explanatory mechanism. Our study, consistent with previous research on camping, finds that individuals are motivated to camp in urban parks as a means of escaping the confinement of their daily lives (93). According to LY9, camping provides a unique context for freedom and escape that fosters both observing others and self-reflection. The surrounding scenery, interactions with others, and various activities act as reflective surfaces mirroring the self. These observational mirrors serve as key mediators for self-observation, reflection, and identification. LY9 notes,

*During camping, I observed individuals living with remarkable refinement and leisure: they basked in the sun on the grass and engaged in lighthearted conversation. In that moment, I felt a profound sense of inner peace and fulfillment*.

Camping, as a distinctive mirror world, evokes individuals' active self-reflection. This unique outdoor experience provides an environment that is markedly different from the hustle and bustle of daily life, allowing people to step back and observe themselves from a fresh perspective. In this natural setting, far removed from the distractions of modern technology and urban routines, campers can engage in deep introspection. They have the opportunity to reconnect with nature, which has been shown to have profound effects on mental and emotional well-being (94).

#### Reconstructing the sense of life's meaning

The complicated reality gradually makes “What is a good life?” vague and difficult to answer. Just as Rosa argued, the sense of disconnection and oppression in modern life cannot be fully eradicated, nor can we directly construct a perfectly free and fulfilling existence. Yet what matters is to seize those genuine moments in lived experience—when we feel truly ourselves, unconstrained and authentically connected (95). In essence, camping is a “non-alienated moment” created by the subject for himself. As an outdoor leisure activity, camping can temporarily isolate the environmental elements of the usual life and provide a way of “contemplation” to re-examine life. As LY2 mentions,

*Only when I camp do I have time to reflect on my losses, possessions, future direction, life plans, professional goals, and family responsibilities*.

Ross finds that in a state of leisure, individuals can reflect on life from a different perspective with a free consciousness (96). When the sense of life's meaning is broken or threatened, people rebuild its framework (97). As an outdoor leisure activity, camping temporarily isolates individuals from daily life and provides a space for contemplation and re-examination of life. Leisurely and comfortable camping practice gives individuals the opportunity to re-appreciate the comfort and authenticity of life, thus forming a positive interaction between individuals and the meaning of life. It also promotes the further sublimation of the life dimension of the healing function of camping.

## Conclusion and discussion

This study demonstrates that camping in urban parks functions as a therapeutic landscape primarily through facilitating a process of self-transformation. Camping in urban parks drives self-transformation through three interdependent stages: escape, reflection, and meaning reconstruction. Escape works not just by disengagement, but by aligning campers' motivations with the campsite's therapeutic features—especially those that draw involuntary attention and ease detachment from daily stress. SHAP analysis confirms Escape as the top predictor of therapeutic outcomes; however, its effects are bidirectional, so optimal escape—enough to create psychological distance, but not so much as to cause discomfort—is essential. Reflection occurs when campers, freed from routine demands, engage peers and surroundings as “reflective surfaces” that support self-observation and introspection. This is backed by the PSYC factor's consistent positive impact: enhanced self-awareness and life appreciation are the measurable signs of reflection. Meaning reconstruction is neither individual achievement nor environmental imposition—it emerges relationally, from congruence between campers and the campsite's social, physical, and symbolic dimensions. SHAP interaction analysis shows the strongest synergy between Compatibility and PSYC, confirming that meaning-making peaks when environmental fit meets psychological realization—proving therapeutic landscapes are co-produced through person–environment resonance, not passively received. By centering camper agency and the transformative process within the therapeutic landscape framework, this research provides a more nuanced understanding of *how* and *why* camping promotes health and well-being, offering valuable insights for theory, campsite design, public health promotion, and future research.

Addressing the booming demand for camping experiences designed to enhance health and well-being, our study emphasizes that camping offers more than mere respite from daily routines; it constitutes a transformative experience that actively promotes health and well-being. Utilizing the therapeutic landscape framework provides a valuable lens for understanding well-being as an emergent outcome of the dynamic interplay between campers and the campsite environment. This aligns with Duff's conceptualization of therapeutic properties as relational achievements rather than fixed attributes of place (98). Consequently, the realization of therapeutic benefits within the specific spatio-temporal context of urban park camping is highly contingent upon individual factors. These include: the camper's level of attentional engagement and restorative experience during camping, their inherent capacity for self-healing, and the degree of alignment between their personal health needs and the camping experience offered. LY7′s contrasting experience vividly illustrates this variability:

*The only overnight camping made me not only not cured but also drove me crazy. I am a poor sleeper, sleeping in the tent, tossing and turning, and even suffering*.

This example underscores that the therapeutic process is fundamentally about forging connections: between humans and their environment, between individuals and their inner selves, and among people. Crucially, therapeutic outcomes are neither singular, guaranteed, nor uniform. They depend significantly on how individuals participate in the camping experience and their embodied encounter with it. Ultimately, the therapeutic potential of camping stems from people's active pursuit of enhanced life quality and personal meaning. Therefore, campsite development must prioritize these public health needs, moving beyond a narrow focus on materialized sensory landscapes toward facilitating meaningful, transformative engagement.

Although this study expands the explanatory perspective of the direct relationship between camping and therapeutic landscape in the Chinese context, there are still some limitations. First, despite adopting a combination of qualitative and quantitative methods, the dataset was confined to one Chinese city. To further enhance the external validity of the findings, future research could incorporate datasets drawn from a wider range of cities. Specifically, 63.01% of respondents were identified as “married with children,” a demographic whose camping motivations are frequently driven by social and familial factors, such as parent-child bonding and shared family experiences. This over representation may introduce a bias that influences the salience of themes related to “profound self-reflection” and “reconstructing the meaning of life,” which were central to our analysis. Future research should aim to include a broader cross-section of campers to validate and extend our findings, ensuring that the psychological benefits of camping are examined across varied demographic contexts. In the questionnaire survey, our study referred to existing mature scales and did not develop relevant scales based on interview data to measure the influence of variables such as “sociality” and “symbolic aspects on the therapeutic outcome of camping. Moreover, we did not account for variations in the camping behavior habits and health concepts between Eastern and Western cultures (99). Future research could further explore cross-cultural comparative analyses to comprehensively deepen our understanding of the experiential mechanisms of urban park camping as therapeutic landscapes. Second, our primary focus was to test the impact of the environments of campsites and individual perceptions on therapeutic outcomes. However, we did not account for the potential cross-domain spillover effects of individual camping experience. Future scholars could further explore these possibilities from other theoretical perspectives. Finally, while camping and therapeutic experiences are continuous and dynamic processes characterized by uncertainty and complexity. In the future, relevant research methods from neuroscience can be introduced to expand the objective and dynamic monitoring of camping and therapeutic landscape experiences.

## Data Availability

The original contributions presented in the study are included in the article/supplementary material, further inquiries can be directed to the corresponding authors.
